# Plasma Lipidomic and Metabolomic Profiling after Birth in Neonates Born to SARS-CoV-2 Infected and Non-Infected Mothers at Delivery: Preliminary Results

**DOI:** 10.3390/metabo11120830

**Published:** 2021-11-30

**Authors:** Aggeliki Kontou, Christina Virgiliou, Thomai Mouskeftara, Olga Begou, Thomas Meikopoulos, Agathi Thomaidou, Eleni Agakidou, Helen Gika, Georgios Theodoridis, Kosmas Sarafidis

**Affiliations:** 1 Department of Neonatology and Neonatal Intensive Care Unit, School of Medicine, Aristotle University of Thessaloniki, Ippokrateion General Hospital, 546 42 Thessaloniki, Greece; angiekon2001@yahoo.gr (A.K.); agathithom@yahoo.com (A.T.); eagaki@hotmail.com (E.A.); 2 Laboratory of Analytical Chemistry, Department of Chemistry, Aristotle University of Thessaloniki, 541 24 Thessaloniki, Greece; cr_virgi@hotmail.com (C.V.); mpegolga@chem.auth.gr (O.B.); thomas_meik@hotmail.com (T.M.); gtheodor@chem.auth.gr (G.T.); 3 Center for Interdisciplinary Research and Innovation (CIRI-AUTH), Balkan Center, B1.4, 10th Km Thessaloniki-Thermi Rd, P.O. Box 8318, 57001 Thessaloniki, Greece; mouskeftara32@gmail.com (T.M.); gkikae@auth.gr (H.G.); 4 Laboratory of Forensic Medicine and Toxicology, School of Medicine, Aristotle University of Thessaloniki, 541 24 Thessaloniki, Greece

**Keywords:** biomarker, COVID-19, transmission, metabolite, newborn

## Abstract

Pregnant women are among the high-risk populations for COVID-19, whereas the risk of vertical transmission to the fetus is very low. Nevertheless, metabolic alternations described in COVID-19 patients may also occur in pregnant women and their offspring. We prospectively evaluated the plasma lipidomic and metabolomic profiles, soon after birth, in neonates born to infected mothers (cases, *n* = 10) and in the offspring of uninfected ones at delivery (controls, *n* = 10). All cases had two negative tests for SARS-CoV-2 (nasopharyngeal swabs) performed 72 h apart. Blood samples were obtained within the first hours after birth. Liquid chromatography-high resolution mass spectrometry (UHPLC-TOF/MS) and gas chromatography-mass spectrometry (GC-MS) were applied for the analyses. Multivariate statistical analysis was performed for data evaluation. Changes in several plasma lipid species-classes (long-chain fatty acids phosphatidylcholines, triglycerides), and amino-acids were identified that allowed for clear discrimination between the study groups. The results of this preliminary investigation suggest that neonates born to Sars-Cov-19 positive mothers, without evidence of viral infection at birth, have a distinct plasma lipidomic and metabolomic profile compared to those of uninfected mothers. Whether these findings are reflective of maternal metabolic alternations due to the virus or a metabolic response following an unidentified neonatal infection warrants further investigation.

## 1. Introduction

The Corona Virus Disease 2019 (COVID-19) pandemic and its cause, Severe Acute Respiratory Syndrome Corona Virus 2 (SARS-CoV-2)—first described in December 2019—are undoubtedly the main protagonists of nowadays.

At the onset of the pandemic and based on previous experience with other respiratory viral infections, pregnant women and neonates were considered at high-risk for SARS-CoV-2, prone to increased morbidity and mortality [1,2,3]. In this context, the scientific community in perinatal−neonatal medicine has made enormous efforts to investigate maternal−fetal and neonatal outcomes. Accumulated evidence shows that pregnancy is an important risk-factor for death, pneumonia, and admission in the intensive care unit in SARS-CoV-2-infected women of reproductive age [4,5].

One of the main scientific questions that arose was the possibility of vertical transmission of the virus from mother to fetus. A recent article co-reporting data on pregnancy and neonatal outcomes of COVID-19 from the United Kingdom (PAN-COVID) and United States (AAP-SONPM) expanded knowledge regarding the issue; in the PAN-COVID registry (which included pregnancies with suspected or confirmed maternal SARS-CoV-2 infection at any pregnancy stage), SARS-CoV-2 positive neonates were reported in 0.9% of all deliveries, and in 2.0% of those with confirmed maternal infection. In the AAP-SONPM registry, 1.8% of the neonates born to mothers tested positive for SARS-CoV-2 14 days before and up to 3 days after delivery were found to be positive to the virus at birth [6]. Other studies combining various diagnostic tests, including placental pathology, showed that vertical transmission of SARS-CoV-2 may indeed be possible, but the likelihood is very small [7]. As documented in a prospective national cohort study from the United Kingdom, neonatal infection in the first 7 days after birth to a mother with perinatal SARS-CoV-2 infection was uncommon, and generally mild or asymptomatic [8]. However, the long-term effects of intrauterine and/or perinatal exposure and the consequences from an apparent or occult SARS-CoV-2 infection to the developing immature neonatal biological systems, and especially the brain, are virtually unknown [9].

Studies on COVID-19 performed so far in neonates have only focused on the epidemiological and clinical characteristics [8,10]. However, metabolomics plays an increasingly important role in the investigation of disease pathogenesis and the development of novel diagnostic−prognostic biomarkers [11] in various disease states, including COVID-19 [12,13]. Therefore, another challenging opportunity using the -omics approach could be the evaluation of the maternal−fetal−neonatal interaction following exposure to SARS-CoV-2 [14], thus clarifying the metabolic effects of the infection on the fetus. Early identification of metabolic disorders in the newborn could help in the prediction of risk for long-term problems, thus allowing for proper monitoring and the timely application of preventive measures.

To the best of our knowledge, no study has been conducted regarding the metabolic profile of neonates born to SARS-CoV-2 positive pregnant women. The only published relevant study that we are aware of referred to the abnormal alterations of breastmilk proteins and metabolites in pregnant women with COVID-19 [15].

We hypothesized that newborns born to mothers with SARS-CoV-2 would have metabolic derangements at birth associated to host/virus interaction. To this aim, we evaluated plasma lipidomic and metabolic profiles, soon after birth, in neonates of SARS-CoV-2 infected mothers and in healthy offspring of uninfected ones.

## 2. Results

### 2.1. Study Population

A total of 20 neonates (10 cases and 10 controls) were studied. Cases included neonates born from nine mothers (one twin gestation); five of them were asymptomatic, two had mild symptoms, and two more had severe manifestations requiring hospitalization and oxygen therapy due to COVID-19. Gestational−perinatal problems were reported in six mothers of cases (gestational diabetes (*n* = 3), placenta previa (*n* = 1), fetal distress (*n* = 1), and monochorionic, diamnotic twins and preeclampsia (*n* = 1)). Delivery was completed via a Caesarian section (*n* = 7) or vaginally (*n* = 2). Five cases were born at term age (≥37 completed weeks of gestation, *n* = 6) and five were premature (<37 weeks of gestation). After birth, four cases developed respiratory problems (respiratory distress syndrome (*n* = 3) and transient tachypnea (*n* = 1)), while one case had feeding intolerance. Examination of the nasopharyngeal swabs (reverse transcription polymerase chain reaction) obtained within the first 12 h of life and at approximately 72 h of life were negative for SARS-CoV-2 in all cases. Controls were born at term age from mothers with single uncomplicated pregnancies, mostly after a vaginal delivery (*n* = 7). Both cases and controls were discharged home.

### 2.2. Metabolomics Analysis

Plasma samples were analyzed by GC-MS to gain information on a wide range of endogenous metabolites with different physicochemical properties, including amino acids, organic acids, carbohydrates, etc., whereas an UHPLC-QTOF/MS method was also applied to obtain information on the lipid species of the samples (lipidomics analysis).

#### 2.2.1. GC-MS Metabolomics Analysis

**Figure 1:**
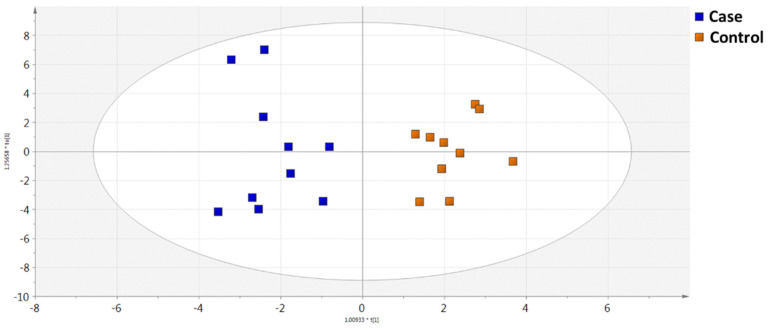
OPLS−DA score plot showing the two groups of samples, case (in blue) vs. control (in yellow) based on the GC-MS analysis data.

Based on the data acquired by the GC-MS methodology, 84 metabolites were identified and further evaluated by multivariate statistical analysis. Data were normalized by the injection standard values and log (base 10) transformed. Principal component analysis (PCA) was performed in order to assess the analytical system’s suitability and reproducibility (QC cluster), as illustrated in Figure S1 (Supplementary Material). The built models revealed discrimination of the two studied groups. In Figure 1, as can be seen, Orthogonal Partial Least Squares Discriminant Analysis (OPLS-DA) score plot provided a statistically significant differentiation between the case and control groups. The model’s validity was cross-verified by R2X, R2Y, and Q2Y values, which were found to be 0.486, 0.891, and 0.632, respectively, as well as by the model’s accuracy (0.85) and CV-ANOVA value (*p* = 0.0.25).

Combining these data with the results from the univariate statistical analysis, five metabolites were found altered in the case samples, namely threonine, threonic acid, glutamine, phenylalanine, and palmitic acid. All of the differentiated metabolites were found to be up-regulated in the case group, except for palmitic acid which was decreased in the case group compared to the control group. Table 1 presents all metabolites found to differ significantly in the plasma of the case group by GC-MS, along with their VIP values, *p* values, area under the curve (AUC) values, and their respective 95% confidence interval (CI) values and logarithm base two of the fold change (FC).

**Figure 2:**
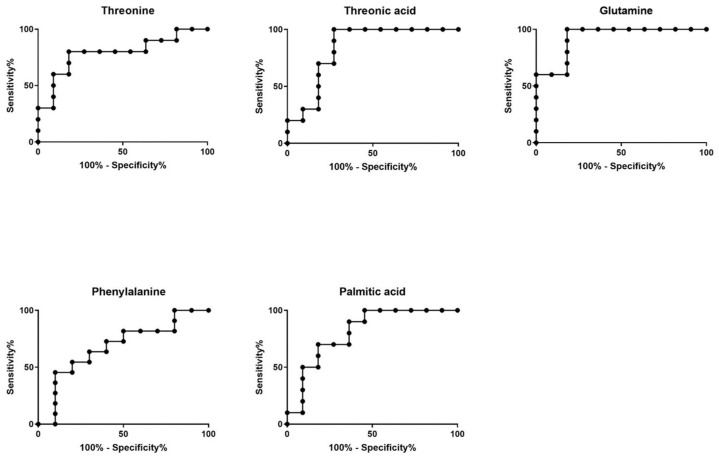
AUC-ROC curves of the significant compounds derived from the GC-MS analysis.

**Figure 3:**
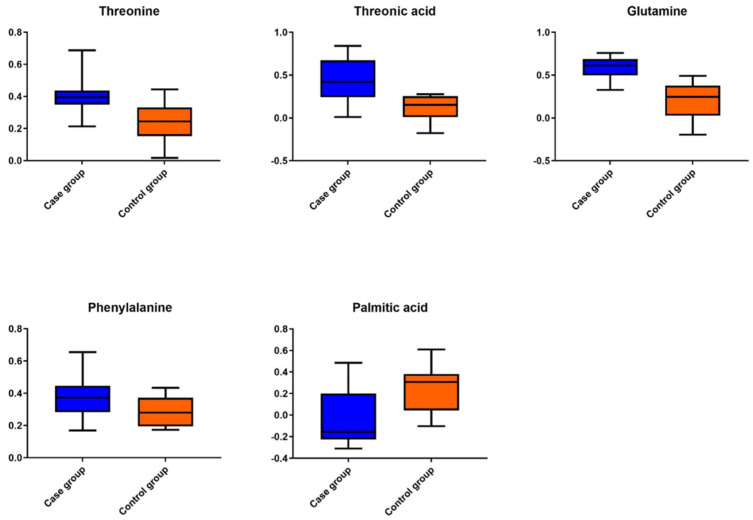
Metabolites that were found to be increased in almost all cases of plasma samples of neonates born to the SARS−CoV−2 positive mother group in comparison to the control group.

Receiver operating characteristic (ROC) curves (Figure 2) and boxplots (Figure 3) for all significant individual metabolites were also constructed. Calculated AUC values were found >0.75 (95% CI > 0.5), as shown in Figure 2, demonstrating the high classification ability of the model to distinguish control and case samples.

#### 2.2.2. UHPLC-TOF/MS Lipidomics Analysis

**Figure 4:**
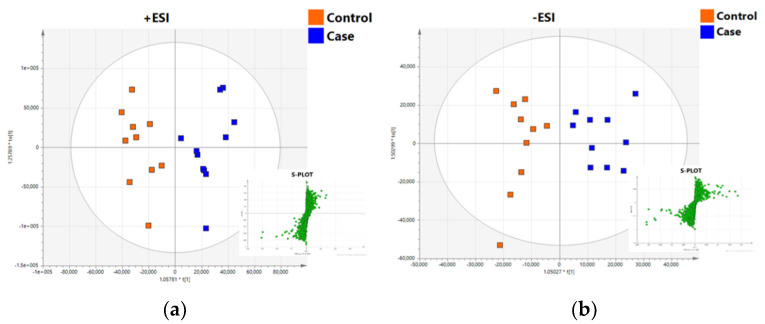
OPLS−DA models showing the classification of cases and control samples based on the UHPLC−TOF/MS lipidomic profile of the plasma samples in (**a**) positive and (**b**) negative ionization modes. On the bottom right S−plot is exhibiting the ions contributing to the classification with increased *p* and *p* (corr).

As stability of the analytical system is essential for efficient and accurate peak alignment and feature identification in untargeted analysis, the detected features were subsequently filtered using accepted and established criteria for QCs, thus only those features with intensities of RSD of less than 30% in the QCs were processed further. From the obtained lipid profiles, a total of 3271 out of the 6456 positive mode ions and 3072 out of the 5481 negative mode ions passed the quality control criteria and were considered for further data analysis. When focusing on the information obtained by the multivariate statistical analysis, QC replicates in the PCA model were clustered together indicating low variability of system’s stability (Supplementary Material Figure S2). The effect of SARS-CoV-2 infection of mothers on the plasma lipid profile of neonates was noticeable and clear clustering could be observed by the OPLS-DA models (Figure 4a,b), which separated the samples into two groups, control (in orange) and cases (in blue). Permutation tests showed that the models were robust, with high predictability (R2Y(cum) and Q2 values of 0.867 and 0.533 for +ESI respectively and 0.795 and 0.453 for −ESI). Cross-validation ANOVA testing (CV-ANOVA) was applied as a significance test of the OPLS models (*p* = 0.02 + ESI, *p* = 0.01 − ESI). The constructed multivariate models, in combination with the univariate statistical analysis, enabled highly significant features to be revealed.

In total, 7 and 25 features in positive and negative ionization modes, respectively, were found by S-plot (see Figure 4a,b) as being statistically significant with *p* ≥ |0.05|, *p*(corr) ≥ |0.5|, and VIP > 1 that passed the filter of *p*-value < 0.05 after performing univariate statistical analysis. The significant features found, and the annotated lipids (MS Level 2, Section 4.7) can be seen in Table 2. From the +ESI data, two lipid classes were found to be affected in the neonate plasma, namely phosphatidylcholines (PCs) and triglycerides (TGs). More specifically, PCs were found to be upregulated, while TGs were decreased in neonates born to Sars-CoV-2 positive mothers. The DDA approach proved highly effective for the identification of lipids in the negative ESI mode. A total of 14 out of 17 compounds were annotated, as can be seen in Table 2. Specifically, fatty acids and PC metabolism seem to be perturbated in neonates of the case group.

**Figure 5:**
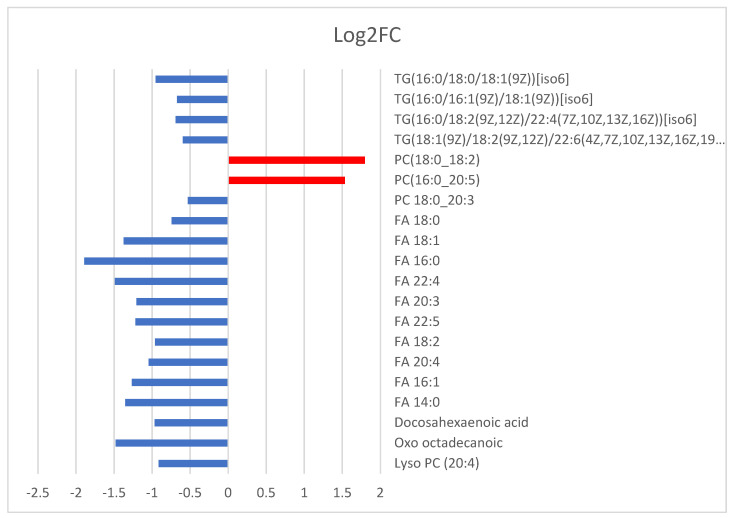
Lipids that were found to be increased or decreased in the plasma of neonates born to SARS-CoV-2 positive mothers.

All the metabolites that were annotated to MSI level 1 or 2 can be seen in Figure 5, plotted in bars based on their Log^2^ fold change values.

**Figure 6:**
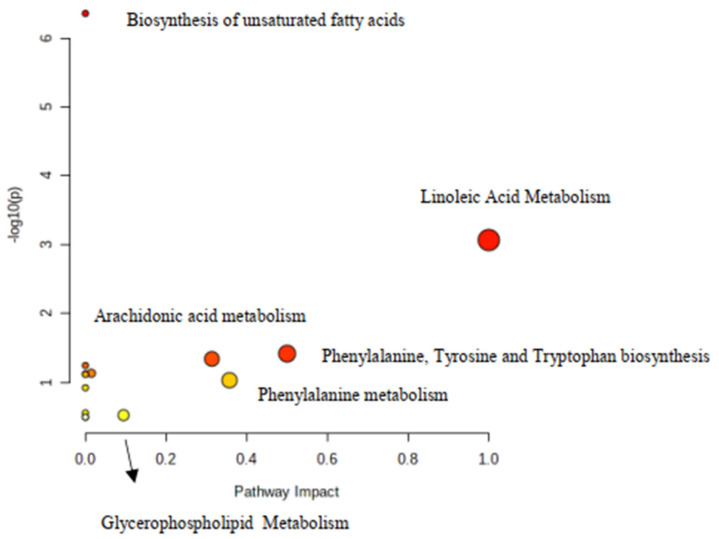
Highly influenced pathways of the significant metabolites as resulted from the online web software MetaboAnalyst 5.0. *p*-values derived from pathway enrichment analysis, whereas pathway impact values from pathway topology analysis. Biochemical pathways that strongly contribute to group differentiation are depicted in bigger and reddish colored cycles.

Metabolic pathways indicated by “MetaboAnalyst” 5.0 to be perturbated based on the lipids and aminoacids that were found to be differentiated are shown in Figure 6.

## 3. Discussion

This cohort, case-control study is the first to report on plasma lipidomic and metabolomic analyses in neonates born to SARS-CoV-2 positive mothers at birth using an untargeted GC-MS and UHPLC-TOF/MS. We found that neonates of SARS-CoV-2 positive mothers, without evidence of viral infection at birth, have significantly altered lipid and amino-acid profiles compared to healthy children born from uninfected mothers.

Lipids are well-known structural components of biological membranes and are an energy source, but they may also act as bioactive signaling molecules. On the other hand, lipids play an essential role in the viral life cycle, including SARS-CoV-2. Viruses, as obligate intracellular pathogens, interact with host cell lipids for viral entry (by mediating fusion or affecting receptor conformation) and viral replication (re-programming of cellular metabolism to remodel lipid membranes and fuel the production of new virions). Further, several classes of lipid mediators may regulate the host immune response to viral infection [16].

Fatty acids (FAs), a major component (Class 3) of lipids, have diverse functions in cells that range from structural “building blocks” of cell membranes to suppliers of energy and signaling molecules. In the present study, neonates born to mothers with COVID-19 had significantly decreased long-chain free fatty acids, including myristic (C14:0), palmitic (C16:0), palmitoleic (C16:1), stearic (C18:0), keto-stearic (oxoC18:0), oleic (C18:1), linoleic (C18:2), eicosatrienoic (C20:3), and arachidonic acid (C20:4), and in very long chain fatty acids (VLCFA) docosatetranoic (C22:4), docosapentanoic (C22:5), and docosahexanoic acid (C22:6). Previous studies provided evidence for the strong involvement of lipids and metabolic dysfunction in COVID-19 [12]. In a large untargeted metabolomic and lipidomic analysis in adults with SARS-CoV-2 infection, several plasma fatty acids, including oleic and arachidonic acids were found to be significantly upregulated in the critically ill patients compared to non-critical ones and healthy controls. Moreover, further analysis showed that the abundance of oleic and arachidonic acids was directly associated with disease severity [12]. Similarly, in a more recent study by Perez-Torres et al. involving patients with severe pneumonia due to SARS-CoV-2, a significant increase of plasma oleic acid and arachidonic acid was documented. In the latter study, however, a decrease in palmitic, palmitoleic, stearic, γ-linoleic, dihomo-γ-linoleic, and eicosapentanoic acid was also observed, compatible with the decrease in total fatty acid [13]. Arachidonic acid (AA) and other unsaturated fatty acids (especially eicosapentaenoic acid (EPA) and docosahexaenoic acid (DHA)) can inactivate enveloped viruses including SARS-CoV-2. As suggested in an opinion article by Das et al., when challenged by microorganisms including viruses such as SARS-CoV-2, immunocytes release AA and other unsaturated fatty acids into their surrounding milieu to inactivate invading organisms, and thus protect tissues. This also implies a higher susceptibility to SARS-CoV-2 infections due to deficiency of AA and other unsaturated fatty acids. It is worth noting that, although some metabolites derived from AA, EPA, and DHA (lipoxins, resolvins, protectins, and maresins) suppress inflammation and enhance healing as well as the function of immunocytes, others produced from AA and EPA (prostaglandins, leukotrienes and thromboxanes have a pro-inflammatory action [17].

Significant changes in cases compared to controls were also observed with respect to additional lipid classes for PCs and TGs. PC comprises 40–50% of the total cellular phospholipids, although different cell types and individual organelles contain distinct phospholipid compositions. Additionally, PC is the predominant lipid class accounting for about 50% of the phospholipids of lung surfactant [18]. In our study, we found a significant up-regulation of PCs (PC (18:0/18:2), PC (16:0/20:5)) and down-regulations of lyso-PC (PC 20:4) in the plasma of cases. Interestingly, Song et al. reported reductions in major classes of plasma glycerophospholipids including PCs, with accompanying increases in their corresponding lysophospholipids, which according to the authors is indicative of an enhanced phospholipase A2 activity in COVID-19 patients [19]. In our study, neonates from COVID-19 positive mothers had reduced TGs as well. A recent lipidomics study showed significant differences in 10 TGs when severe and mild patients were compared each other, specifically five TGs increased in the serum of COVID-19 hospitalized patients, and five decreased in those with moderate and mild disease [20]. Additionally, a quantitative lipidome and metabolome study profiling in adults with mild to severe COVID-19 and healthy controls showed reduced neutral lipids including medium- and long-chain TGs in subjects with COVID-19 [19].

In line with studies in adults with COVID-19, we observed significant changes in amino-acids; cases were found to have significantly elevated plasma levels of l-glutamine, l-threonine, and l-phenylalanine compared with the controls. Glutamine seems to play an important role in COVID-19, but the reported alternations of the specific essential amino-acid in diseased patients were controversial, either increased [12] or decreased [21,22]. Interestingly, reduced glutamine levels were negatively correlated with several laboratory markers of disease severity such as lactate dehydrogenase, C reactive protein, and blood oxygen levels [22]. In line with our results, increased phenylalanine levels have also been reported in COVID-19 patients [21,22]. Elevated circulating levels of aromatic amino acids such as phenylalanine are consistent with liver dysfunction and the inability to catabolize large amounts of aromatic amino acids following the breakdown of endogenous proteins.

The evaluation for the first time of the lipidomic and metabolic profiles of newborn infants born to infants of COVID-19 positive mothers at delivery, either asymptomatic or symptomatic, is the novelty and advantage of the study. An important question, however, that is raised in the present study is whether lipid and amino-acid alternations observed in cases were of a maternal origin and passive transportation to the fetus before delivery, or if they reflect a fetal host response to an unidentified infection due to the virus. SARS-CoV-2 spike protein may cross the placental barrier and, therefore, induce an immune response to the fetus [9]. However, irrespective whether the source of inflammation is direct (e.g., when the fetus-neonate is positive for SARS-CoV-2) or indirect (from the maternal circulation, especially in the presence of “cytokine storm”), inflammation represents an important risk-factor for later neurodevelopmental disorders [9]. Of note, there are no metabolomic studies in pregnant women with SARS-CoV-2. The paired maternal−neonatal lipidomic and metabolomic profiling along with the assessment of inflammatory indicators would be helpful in clarifying the aforesaid issues. Moreover, the rarity of vertical transmission and neonatal infection at birth may explain why we were not able to study neonates positive for SARS-CoV-2 or suffering COVID-19. In this case, metabolic alternations could be different and more severe, but this is a hypothesis that warrants future investigation. The evolution of metabolome and lipidome over time is another interesting point. Most probably, lipid and amino-acid changes would gradually resolve within weeks or months, but this speculation could not be tested herein due to the study design. Lastly, four of our cases exhibited respiratory morbidities, which may have also influenced their lipidomic and metabolomic profiles.

## 4. Materials and Methods

### 4.1. Study Population

The study protocol was approved by the ethics committee (Scientific Council) of Hippokration General Hospital. Written consent was obtained from all parents prior to the enrollment of their neonates in the study. However, when the medical condition of the mother did not allow her to have the consent signed, parental consent only was permitted.

This prospective, cohort, case-control, pilot study was performed in a single, tertiary Neonatal Intensive Care Unit (NICU) during the SARS CoV-2 pandemic in Greece. Eligible participants for the study were preterm and term neonates born to mothers with confirmed COVID-19 at delivery (cases) and healthy ones born to SARS CoV-2 mothers (controls). Exclusion criteria included maternal history of SARS CoV-2 infection before delivery and neonatal problems such as perinatal asphyxia, known congenital malformations, kidney and liver dysfunction, and early-onset sepsis.

### 4.2. Samples

Blood samples were collected from cases and controls during the first 12 h as part of their evaluation for a neonatal problem or for the purpose of the present study. Blood was collected in microtubes containing K2 EDTA (Becton, Dickinson and Company, Franklin Lakes, NJ, USA) and transferred at the hospital’s central laboratory for centrifugation (and examination as required). Obtained plasma was stored at −80 °C until further analysis.

### 4.3. Chemicals and Reagents

Methanol (MeOH), acetonitrile (ACN), formic acid (FA), methyl tert-butyl ether (MTBE) and iso-propanol (IPA) (LC-MS grade) were obtained from CHEM-LAB NV (Zedelgem, Belgium). Ammonium formate was purchased from PANREAC QUIMICA SLU (Barcelona, Spain). Deionized water was obtained from a Milli-Q ultra-pure grade water system (18 MΩ∙cm^−1^) (Millipore, Bedford, MA, USA) (LC-MS grade). Methoxamine hydrochloride (MeOX), N-Methyl-N-(trimethylsilyl)trifluoroacetamide (MSTFA), N,O-Bis(trimethylsilyl)trifluoroacetamide (BSTFA), trimethylchlorosilane (TMCS), pyridine anhydrous were obtained from Sigma-Aldrich (Merck, Darmstadt, Germany). Myristic acid-d_7_ and pentadecane were purchased from Sigma-Aldrich (Merck Darmstadt, Germany).

### 4.4. Sample Preparation

For untargeted GC-MS analysis, 150 μL of ice cold MeOH and 10 μL of myristic acid-d_7,_ 100 mg/L (Internal standard) were added at 50 μL of plasma samples to extract a plethora of various metabolites. The mixture was vortex-mixed for 5 min and then centrifuged at 11,200× *g* for 10 min. Supernatant (160 μL) was transferred and evaporated to dryness. Subsequently, 50 μL of MeOX 2% pyridine, were added and shaken for 1 min and then transferred in a heating device for 2 h at 60 °C (first derivatization step). After that, a second derivatization step took place by adding 100 μL of MSTFA 1% TMCS and left for 1 h at 60 °C. At last, 10 μL of pentadecane (100 mg/L) were inserted to the sample, as injection standard. A Quality Control sample (QC) was prepared by mixing equal volumes of each sample and QC analysis was performed in three replicates.

For UHPLC-TOF/MS lipidomics analysis, 50 µL of plasma samples were treated with 700 µL of the organic solvent mixture MTBE:MeOH (3:1 *v*/*v*), followed by 5 min vortex (20 Hz). The samples were then centrifuged for 30 min at 4 °C and 25,200× *g*. Supernatants (600 µL) were transferred to Eppendorf tubes and evaporated to dryness (SpeedVac, Eppendorf Austria GmbH, Wien, Austria) followed by reconstitution with 150 μL H_2_O:ACN:IPA (1:1:3 *v*/*v*). A pooled sample (QC) was prepared as representative by mixing equal volumes of each plasma sample. Diluted QCs (1:2, 1:4, 1:6, 1:8 in H_2_O:ACN:IPA (1:1:3)) were also analysed in order to evaluate dilution integrity of the detected features.

### 4.5. GC-MS Analysis

GC-MS analysis was performed on an Agilent Technologies 7890A GC, coupled with a 5975C MSD (Agilent Technologies, Santa Clara, CA, USA), equipped with a PTV injector and a CTC autosampler. Chromatographic separation was carried out on a 30-m HP-5 ms UI (Agilent J&W) capillary column, with a film thickness of 0.25 μm and an i.d. of 0.25 μm, equipped with a secondary 1.5-m deactivated Agilent column with a film thickness of 0.18 mm, to perform back-flush elution. The duration of the analysis was 30 min, with a 12 min back-flush step. The temperature program was as follows: the initial column temperature was maintained at 60 °C for 1 min and increased to 300 °C with a 10 °C/min rate, where the temperature was held for 6 min. Helium (purity 99.999%) was used as the carrier gas at a constant flow rate of 3 mL/min. An injection volume of 1 μL was performed in splitless mode. The PTV injector temperature was set at 110 °C with a ramp of 720 °C/min to 270 °C for 1 min and then to 350 °C for 5 min. The mass spectrometer (MS) was operated at the electron impact ionization mode (EI, 70 eV, ion source temperature 230 °C, transfer line temperature 250 °C). The mass spectra were acquired over the range of 50–600 amu in full scan mode, with a solvent delay of 6 min.

### 4.6. UHPLC-TOF/MS Analysis

Chromatographic separations were performed on a UHPLC Elute system with an ACQUITY UPLC CSH C18 (2.1 × 100 mm, 1.7 μm *p*/n 186005297) column (Waters Ltd., Elstree, UK). The mobile phase system consisted of solvent A: ACN:H_2_O (60:40 *v*/*v*), 10 mM ammonium formate, and 0.1% formic acid, and solvent B: IPA:ACN (90:10 *v*/*v*) and 0.1% formic acid. Separation was performed at 55 °C with a flow rate of 0.4 mL/min using the following gradient elution program: 60–57% A(0.0–2.0 min), 57–50% A (2.0–2.1 min; curve 1), 50–46% A(2.1–12.0 min), 46–30% A (12.0–12.1 min; curve 1), 30–1%A (12.1–18 min), 1–60% A (18.0–18.1 min), and 60% A (18.1–20.0 min). A cycle with a strong wash solvent IPA:ACN (90:10 *v*/*v*) (1000 μL) and a cycle with weak wash solvent (1000 μL) ACN:H_2_O (60/40 *v*/*v*) was performed before and after each injection. The injection volume was set at 5 and 10 μL in positive and negative mode, respectively. The autosampler temperature was set at 8 °C. The MS data were acquired using a TIMS TOF mass spectrometer (Bruker, Billerica, MA, USA) in positive and negative ionization mode using the following settings in ESI: capillary ± 4.2 kV, dry temperature 200 °C, dry gas 10 L/min, and nebulizer gas 2 Bar. The ion transfer parameters were optimized, Funnel RF 1 and 2 were set to 250 Vpp, Multipole RF at 200 Vpp and deflection delta at 80 V. Quardrupole parameters, ion energy, and low mass were set to 5 eV and 200 *m*/*z* respectively. With regards to collision cell, collision energy and collision RF were set to 10 eV and 1100.0 Vpp, respectively, applied ions’ transfer time was 54 µs and pre pulse storage 5 µs.

MS/MS experiments both DDA (data-based acquisition) and with no precursor ion selection were performed (bbCID). With regards to bbCID, collision energy for low energy was set to 10, and for high energy, to 20−50, spectra rate was set to 3 Hz. MS/MS data scans were acquired for QC samples and a randomly selected sample from each group. Auto MS/MS was applied using Dynamic MS/MS spectra acquisition with 6 and 10 Hz as the minimum and maximum spectra rates, respectively. Collision energy was set at 30 for all precursor ions and 0.5 s applied for cycle time. Calibrant (sodium formate, 10 mM) was infused to MS during 0.1–0.3 s with flow rate 60.000 µL/h.

With regards to the analytical sequence, the samples were analyzed in one batch for each ionization mode, initially three blank samples (H_2_O:ACN:IPA 1:1:3 *v*/*v*), followed by five QC samples were analyzed before the analytical sequence to achieve system equilibration. QC samples were analyzed every after five samples.

### 4.7. Data Processing and Statistical Analysis

Chromatographic GC-MS data were treated by MSD CHEMSTATION software and NIST17 Mass Spectral Library (*mainlib* library). Free online Assignment Validator and Integrator (GAVIN) script for MATLAB (MathWorks, Natick, MA) was also used as a complement to AMDIS (NIST) for data processing. Initially, chromatographic peak deconvolution and identification was performed by AMDIS, applying simple mode with a minimum match factor of 50% and using *mainlib* library for compounds’ identification. AMDIS results files and raw data files (.cdf) were further processed by GAVIN3 software to enable, among other tasks, peak alignment [23]. Chromatographic peak areas of Extracted Ion Chromatograms (EICs) together with possible identities and retention times were exported from MATLAB.

After acquisition of high-resolution analysis by UHPLC-TOF/MS, centroided data were recalibrated using sodium formate clusters. Files were converted to mzML by MSConvert (ProteoWizard 3.0.11567). Chromatographic peak detection, retention time alignment, and feature grouping were carried out using XCMS (version 3.2.0) in the R programming environment. Values were reported as peak intensity (area) [24]. Data were further processed using both Microsoft Excel and the advanced statistical software GraphPad Prism 7.0 for Windows (GraphPad Software, La Jolla, CA, USA). Data were assessed for normal distribution based on D’Agostino-Pearson (95% de) statistical test. Two-tailed *t*-test, with unequal variance and a threshold of *p* < 0.05, was performed. Area under the curve–receiver operating characteristic (AUC–ROC) curves and box plots were illustrated for the significant metabolites and lipids, while the logarithm base two of fold change (log2FC) were calculated as the ratio of the final value (case groups) over the initial value (control group).

The SIMCA package (version 13.0.2.; Umetrics, Sweden) was used for multivariate statistical analysis and biomarker assessment via Variable Importance for the Projection plots (VIP), loading plots, S-plots, *p*(corr), and hotelling’s lines [25]. Principal components analysis (PCA) and orthogonal projection to latent structures discriminant analysis (OPLS-DA) were performed to assess data in a multivariate setting. Models validation was evaluated via e cross-validation parameters (R2X: fraction of the variation in X explained by the model, R2Y: total sum of variation in Y explained by the model and Q2Y: goodness of prediction), permutation plots and CV-ANOVA pvalue. Biochemical pathways analysis was investigated using the online available intelligent platform “MetaboAnalyst 5.0” [26].

For lipid structure assignment, accurate *m*/*z* measurements of statistically significant features were first matched to metabolites from online MS databases (Metlin, HMDB and Lipidmaps). After an assessment of the retention time and isotopic pattern, tandem MS fragmentation pattern (from DDA, MS/MS experiments) was employed for further structural elucidation; spectra were compared with those from online available databases and literature (where available). Assignment according to fragmentation pattern was dependent on the ability to obtain spectra with adequate signal. Based on the identification level system of metabolites from untargeted analysis identification, “Level 2” was reached for most of the ions [27]. Network and path analysis was performed with MetPA (http://www.metaboanalyst.ca, accessed on 1 October 2021) a web-based tool dedicated to the analysis and visualization of metabolomic data within the biological context of metabolic pathways.

## 5. Conclusions

We investigated the effect of maternal SARS-CoV-2 infection on the plasma lipidomic and metabolomic profiles of their offspring at birth using untargeted GC-MS and UHPLC-TOF-MS. The results of this preliminary investigation suggest that neonates born to Sars-Cov-19 positive mothers, without evidence of viral infection at birth, have a distinct plasma lipidomic and metabolomic profile compared to those of uninfected mothers. Whether these findings are indirectly linked to metabolic alternations of the mothers due to the viral infection or directly to a fetal-neonatal response against the virus needs further investigation.

## Figures and Tables

**Table 1:** Statistically significant metabolites revealed by the GC-MS analysis after statistical analysis and comparison of the examined groups. VIP values, *p*-value, area under the curve (AUC), and their respective 95% confidence interval (CI) values and logarithm base two of the fold change (FC) are given.

Compound	VIP	*p* Value	AUC-ROC (95% CI)	Log2FC
Threonine	1.9	9.6 × 10^−3^	0.79 * (0.59–0.99)	0.55
Threonic acid	2.1	4.0 × 10^−3^	0.84 ** (0.65–1.0)	1.16
Glutamine	2.5	3.9 × 10^−4^	0.93 *** (0.82–1.0)	1.15
Phenylalanine	1.6	3.0 × 10^−2^	0.75 (0.45–0.92)	0.4
Palmitic acid	1.8	1.2 × 10^−2^	0.81 * (0.62–1.0)	−0.85

AUC-ROC *p*-value: * < 0.05, ** < 0.005, and *** < 0.0005.

**Table 2:** Features found to differ significantly by ±ESI UHPLC-TOF/MS in the plasma of neonates born to SARS-CoV-2 positives mothers in comparison with the controls.

A/A	VIP	*m*/*z*	RT min	*p* Value	Log2FC	Annotation
**+ESI**						
1	9	780.557	5.2	2.18 × 10^−2^	1.53	PC (16:0_20:5)
2	11	788.619	8.6	7.12 × 10^−3^	1.80	PC (18:0_18:2)
3	3	824.619	9.1	2.89 × 10^−3^	−1.58	Unknown
4	3	946.783	15.3	1.15 × 10^−2^	−0.59	TG(18:1(9Z)/18:2(9Z,12Z)/22:6(4Z,7Z,10Z,13Z,16Z,19Z))[iso6]
5	4	925.802	15.7	2.86 × 10^−2^	−0.69	TG(16:0/18:2(9Z,12Z)/22:4(7Z,10Z,13Z,16Z))[iso6]
6	12	848.771	15.7	1.21 × 10^−3^	−0.67	TG(16:0/16:1(9Z)/18:1(9Z))[iso6]
7	11	878.819	16.3	4.29 × 10^−4^	−0.95	TG(16:0/18:0/18:1(9Z))[iso6]
**−ESI**						
1	5	383.149	0.6	7.23 × 10^−5^	−1.09	Unknown
2	3	411.180	0.6	3.92 × 10^−3^	−0.78	Unknown
3	4	588.324	1.0	6.23 × 10^−3^	−0.91	Lyso PC (20:4)
4	3	297.240	1.0	1.36 × 10^−4^	−1.48	Oxo octadecanoic
5	5	327.229	1.8	7.26 × 10^−3^	−0.97	Docosahexaenoic acid
6	3	227.198	1.9	1.11 × 10^−4^	−1.35	FA 14:0
7	10	253.215	2.0	8.96 × 10^−4^	−1.26	FA 16:1
	4	254.217	2.0	8.87 × 10^−4^	−1.26	
8	7	303.229	2.0	2.46 × 10^−3^	−1.05	FA 20:4
	3	304.232	2.0	2.94 × 10^−3^	−0.95	
9	3	280.232	2.1	3.53 × 10^−3^	−0.96	FA 18:2
10	3	329.245	2.3	3.19 × 10^−3^	−1.22	FA 22:5
11	3	305.245	2.3	3.24 × 10^−4^	−1.20	FA 20:3
12	3	445.328	2.5	1.44 × 10^−2^	−1.04	Unknown
13	4	331.260	2.6	4.65 × 10^−4^	−1.49	FA 22:4
14	3	255.247	2.7	3.62 × 10^−4^	−1.89	FA 16:0
	7	256.233	2.7	6.80 × 10^−5^	−1.09	
	17	255.230	2.7	3.70 × 10^−4^	−1.14	
15	5	281.264	2.8	6.85 × 10^−3^	−1.37	FA 18:1
	8	282.248	2.8	2.84 × 10^−3^	−0.84	
	13	281.246	2.8	2.25 × 10^−2^	−0.65	
16	4	284.264	3.6	8.83 × 10^−5^	−0.74	FA 18:0
	9	283.261	3.6	6.09 × 10^−4^	−0.85	
17	4	857.598	9.3	1.24 × 10^−2^	−0.32	PC 18:0_20:3
	6	856.593	9.3	3.22 × 10^−3^	−0.53	
**+ESI**						
1	9	780.557	5.2	2.18 × 10^−2^	1.53	PC (16:0_20:5)
2	11	788.619	8.6	7.12 × 10^−3^	1.80	PC (18:0_18:2)
3	3	824.619	9.1	2.89 × 10^−3^	−1.58	Unknown
4	3	946.783	15.3	1.15 × 10^−2^	−0.59	TG(18:1(9Z)/18:2(9Z,12Z)/22:6(4Z,7Z,10Z,13Z,16Z,19Z))[iso6]
5	4	925.802	15.7	2.86 × 10^−2^	−0.69	TG(16:0/18:2(9Z,12Z)/22:4(7Z,10Z,13Z,16Z))[iso6]
6	12	848.771	15.7	1.21 × 10^−3^	−0.67	TG(16:0/16:1(9Z)/18:1(9Z))[iso6]
7	11	878.819	16.3	4.29 × 10^−4^	−0.95	TG(16:0/18:0/18:1(9Z))[iso6]
**−ESI**						
1	5	383.149	0.6	7.23 × 10^−5^	−1.09	Unknown
2	3	411.180	0.6	3.92 × 10^−3^	−0.78	Unknown
3	4	588.324	1.0	6.23 × 10^−3^	−0.91	Lyso PC (20:4)
4	3	297.240	1.0	1.36 × 10^−4^	−1.48	Oxo octadecanoic
5	5	327.229	1.8	7.26 × 10^−3^	−0.97	Docosahexaenoic acid
6	3	227.198	1.9	1.11 × 10^−4^	−1.35	FA 14:0
7	10	253.215	2.0	8.96 × 10^−4^	−1.26	FA 16:1
	4	254.217	2.0	8.87 × 10^−4^	−1.26	
8	7	303.229	2.0	2.46 × 10^−3^	−1.05	FA 20:4
	3	304.232	2.0	2.94 × 10^−3^	−0.95	
9	3	280.232	2.1	3.53 × 10^−3^	−0.96	FA 18:2
10	3	329.245	2.3	3.19 × 10^−3^	−1.22	FA 22:5
11	3	305.245	2.3	3.24 × 10^−4^	−1.20	FA 20:3
12	3	445.328	2.5	1.44 × 10^−2^	−1.04	Unknown
13	4	331.260	2.6	4.65 × 10^−4^	−1.49	FA 22:4
14	3	255.247	2.7	3.62 × 10^−4^	−1.89	FA 16:0
	7	256.233	2.7	6.80 × 10^−5^	−1.09	
	17	255.230	2.7	3.70 × 10^−4^	−1.14	
15	5	281.264	2.8	6.85 × 10^−3^	−1.37	FA 18:1
	8	282.248	2.8	2.84 × 10^−3^	−0.84	
	13	281.246	2.8	2.25 × 10^−2^	−0.65	
16	4	284.264	3.6	8.83 × 10^−5^	−0.74	FA 18:0
	9	283.261	3.6	6.09 × 10^−4^	−0.85	
17	4	857.598	9.3	1.24 × 10^−2^	−0.32	PC 18:0_20:3
	6	856.593	9.3	3.22 × 10^−3^	−0.53	

## Data Availability

Not applicable.

## Supplementary Materials

The following are available online at https://www.mdpi.com/article/10.3390/metabo11120830/s1, Figure S1: PCA score plot showing the examined groups together with QC samples based on the GC-MS analysis data. QC samples are clustering together, showing system’s stability, Figure S2: Indicative PCA score plot showing the examined groups together with QC samples based on the UHPLC-TOF/MS (+ESI) lipidomic profile of the plasma samples. QC samples are clustering together, show-ing system’s stability.
