# Supplementary material for: Plasma Lipidomic and Metabolomic Profiling after Birth in Neonates Born to SARS-CoV-2 Infected and Non-Infected Mothers at Delivery: Preliminary Results
Source: Metabolites. 2021 Nov 30;11(12):830. doi: 10.3390/metabo11120830 (PMC8706054; doi:10.3390/metabo11120830)
Supplement: Supplementary file 1 [file metabolites-11-00830-s001.zip › metabolites-1470998-SI.pdf]

Supplementary Material

Plasma lipidomic and metabolomic profiling after birth in neonates born to sars-cov-19 infected and non-infected mothers at delivery: preliminary results

Aggeliki Kontou[1‡], Christina Virgiliou[2,4‡], Thomai Mouskeftara[3,4], Olga Begou[2,4], Thomas Meikopoulos[2,4], Agathi Thomaidou[1], Eleni Agakidou[1], Helen Gika[3,4], Georgios Theodoridis[2,4], Kosmas Sarafidis[1]

[1] 1st Department of Neonatology and Neonatal Intensive Care Unit, School of Medicine, Aristotle University of Thessaloniki, Ippokrateion General Hospital, Thessaloniki, Greece; angiekon2001@yahoo.gr; agathithom@yahoo.com; kosmas.sarafidis@gmail.com
[2] Laboratory of Analytical Chemistry, Department of Chemistry, Aristotle University of Thessaloniki, Greece; cr_virgi@hotmail.com; mpegolga@chem.auth.gr; thomas_meik@hotmail.com; gtheodor@chem.auth.gr
[3] Laboratory of Forensic Medicine and Toxicology, School of Medicine, Aristotle University of Thessaloniki, Greece; mouskeftara32@gmail.com; gkikae@auth.gr
[4] Center for Interdisciplinary Research and Innovation (CIRI-AUTH), Thermi GR 57001, Greece
[‡] Authors with equal contribution
* Correspondence: K.S.: kosmas.sarafidis@gmail.com

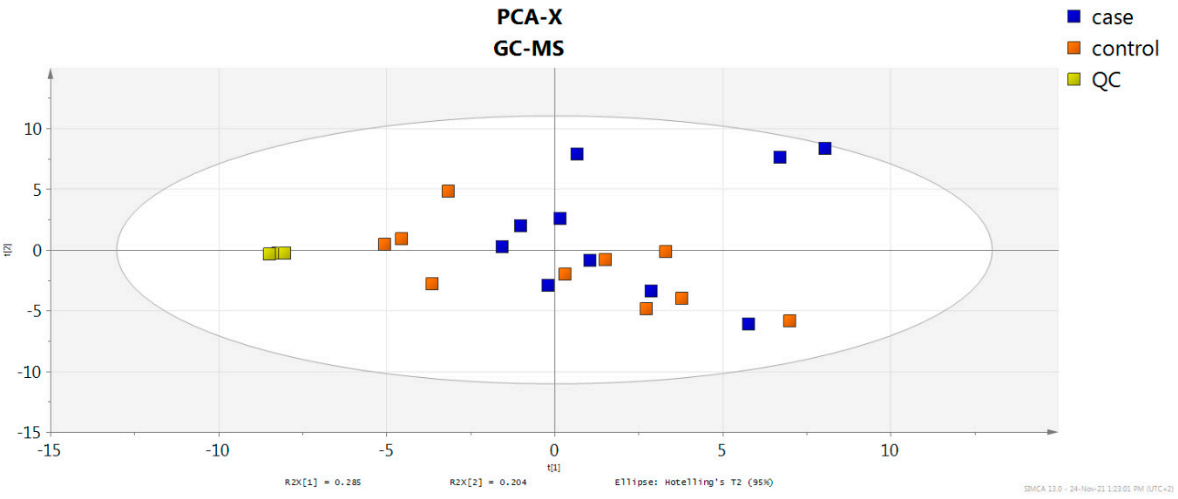

Figure S1: PCA score plot showing the examined groups together with QC samples based on the GC-MS analysis data. QC samples are clustering together, showing system's stability.

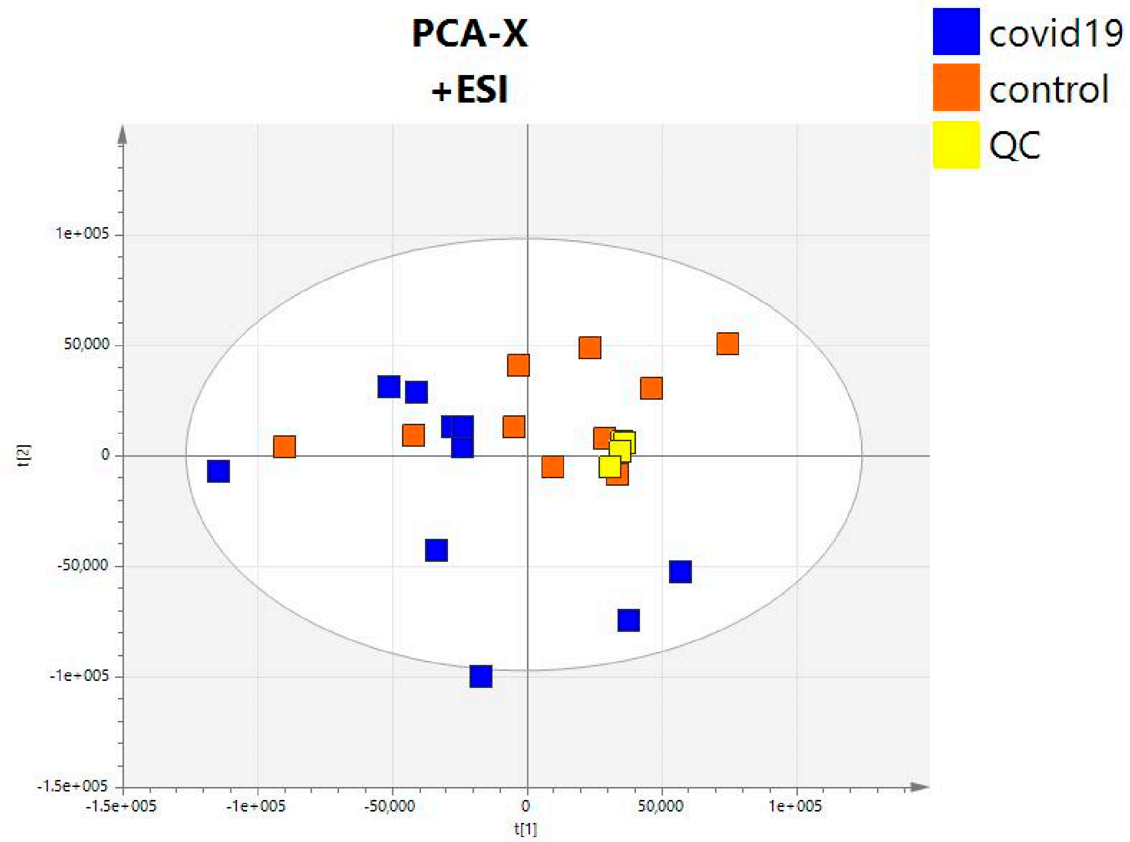

Figure S2: Indicative PCA score plot showing the examined groups together with QC samples based on the UHPLC-TOF/MS (+ESI) lipidomic profile of the plasma samples. QC samples are clustering together, showing system's stability.
